# Human Cytomegalovirus Suppresses Estrogen and Progesterone Receptor Expression in Hormone Receptor-Positive Breast Cancer Cells: Implications for Endocrine Resistance

**DOI:** 10.3390/cancers18152519

**Published:** 2026-08-06

**Authors:** Erica C. Garcia, Ian J. LaRue, Nathan D. Griggs, Juliet V. Spencer

**Affiliations:** 1 Division of Biology, Texas Woman’s University, Denton, TX 76204, USA; ecg4003@med.cornell.edu (E.C.G.); larue_i@yahoo.com (I.J.L.);; 2 Department of Pediatrics, Weill Cornell Medicine, New York, NY 10065, USA; 3 School of Natural & Environmental Sciences, College of Charleston, Charleston, SC 29424, USA

**Keywords:** breast cancer, estrogen receptor-α (ER-α), hormone receptor (HR), hormone therapy, Human Cytomegalovirus (HCMV), Human Herpesvirus 5, progesterone receptor (PR)

## Abstract

Background: Hormone receptor-positive breast cancer depends on estrogen and progesterone signaling, yet factors that modulate hormone receptor expression within tumors remain incompletely understood. Human cytomegalovirus (HCMV), a widespread herpesvirus detected in breast tumors, has been associated with reduced expression of estrogen receptor-α (ERα) and progesterone receptor (PR), but a direct causal relationship has not been established. Methods: ER+/PR+ breast cancer cell lines MCF-7 and T47D were infected with HCMV in vitro. ERα and PR protein levels were assessed by immunoblotting, and transcript levels of *ESR1* and *PGR* were quantified by qPCR. To determine whether virus replication was required, parallel experiments used UV-inactivated HCMV. Results: HCMV infection resulted in a marked reduction in ERα and PR protein levels in both cell lines, accompanied by decreased *ESR1* and *PGR* transcript levels by 48 h post-infection. Notably, UV-inactivated HCMV produced a comparable suppression of hormone receptor expression, indicating that viral gene expression and productive replication are not required for this effect. Conclusions: These findings indicate that HCMV exposure suppresses ERα and PR expression in breast cancer cells through a replication-independent mechanism. This effect suggests that viral components or host responses to infection may alter hormone receptor signaling within tumors, with potential implications for hormone receptor signaling and endocrine therapy responsiveness that warrant further investigation. Together, these results identify HCMV as a previously underrecognized modulator of hormone receptor pathways in breast cancer.

## Simple Summary

Breast cancer is often classified based on the presence of hormone receptors that allow tumors to respond to treatments targeting estrogen and progesterone signaling. However, some tumors lose these receptors over time, making them harder to treat. In this study, we examined whether exposure to human cytomegalovirus, a common virus found in many people, could directly affect hormone receptor levels in breast cancer cells. Using laboratory models, we found that viral exposure reduced the expression of key hormone receptors, even when the virus was unable to replicate. These results suggest that viral components or the cellular response to infection may influence how breast cancer cells respond to hormone-based therapies. This work highlights a potential new factor contributing to variability in tumor behavior and treatment outcomes, and it may help guide future research on virus-associated changes in cancer biology.

## 1. Introduction

Breast cancer is the most commonly diagnosed cancer in women [1]. In fact, it is estimated that about one in eight women will develop breast cancer in their lifetime according to the American Cancer Society [1]. Recent evidence suggests that Human Cytomegalovirus (HCMV) may influence breast cancer progression [2,3,4]. HCMV is a widespread member of the *Herpesviridae* family that infects a wide range of cell types, including endothelial cells, epithelial cells, fibroblasts, and monocytes [5,6], all of which are components of the tumor microenvironment. Herpesviruses such as HCMV are ancient and thus are likely powerful manipulators of cell biology. With a genome of 230 kb in length and with 170 open reading frames [7], HCMV possesses numerous mechanisms for immune evasion and control of intracellular processes [8], including methods of altering cellular morphology for increased virus production [9,10], and changing cellular metabolism to cope with increased demand for energy and viral particle building materials [11,12,13]. These processes that occur in infected cells may inadvertently complement the needs of cancer cells, which need to escape immune detection and meet metabolic demands for enhanced proliferation. Cancer cells can therefore benefit from these byproducts of infection, which may lead to a more invasive phenotype.

HCMV can establish latent or persistent infection in the breast and is well recognized to reactivate during lactation, resulting in viral shedding in breast milk [14]. This demonstrates that breast tissue can serve as a site of viral persistence and active replication. Several studies have reported detection of HCMV proteins and nucleic acids in breast tumors [2,3,15], with some studies observing higher detection rates in tumor tissue than in adjacent normal breast tissue [15]. However, the reported prevalence of HCMV in breast cancer has varied considerably among patient cohorts and laboratories [16,17]. This variability likely reflects differences in patient populations, tissue processing, viral burden, and detection methodologies, as well as the focal and often low-level nature of HCMV infection within tumors. Despite these differences, multiple independent studies have identified HCMV within subsets of breast tumors, supporting continued investigation of its potential biological significance [2,3,15,18]. Additionally, in areas where high levels of viral protein were detected, significantly lower levels of the hormone receptors (HR) estrogen receptor-α (ERα) and progesterone receptor (PR) were found [18]. This finding is significant for breast cancer patients, because immunohistochemistry testing for hormone receptors is among the first evaluations performed on breast tumor biopsies, and influences the patient’s course of treatment [19]. HR-positive breast tumors make up a significant proportion of all breast cancer diagnoses and can be treated using endocrine therapy, which targets HR signaling [19]. Because of the availability of a molecular target on these tumor cells, HR-positive breast cancer often carries the best prognosis [19]. However, triple negative breast tumors with extremely low levels of HRs are not responsive to endocrine therapy and tend to be more aggressive [20]. Therefore, the negative correlation between viral protein and HR levels is highly clinically relevant. However, whether HCMV directly modulates hormone receptor expression in breast cancer cells, and whether this effect depends on active viral replication, remains unclear. To investigate this possibility, we infected two HR-positive breast cancer cell lines, MCF-7 and T47D, with HCMV. We sought to determine whether HCMV infection causes changes in HR levels and to identify what cellular mechanisms might underlie this phenomenon.

## 2. Materials and Methods

### 2.1. Cells, Chemicals, and Viruses

MCF-7 mammary epithelial cells originating from adenocarcinoma (ATCC, Manassas, VA, USA, Catalog # HTB-22) and T47D mammary epithelial cells originating from ductal carcinoma (ATCC, Catalog # HTB-133) were cultured at 37 °C in Dulbecco’s modified Eagle media (DMEM) with L-glutamine, 4.5 g/L glucose and sodium pyruvate (Corning, Manassas, VA, USA, Catalog # MT10013CV), which was supplemented with 10% fetal bovine serum, non-essential amino acids, and HEPES solution (4-(2-hydroxyethyl)-1-piperazineethanesulfonic acid). Adult retinal pigment epithelial (ARPE-19) cells of normal epithelial origin (ATCC, Catalog # CRL-2302) were used for virus propagation. Neonatal Foreskin Fibroblasts (NuFF) were obtained from Global Stem (Rockville, MD, USA, Catalog # GSC-3002) and used for plaque assays to determine virus titer. All cells were grown in a humidified chamber in the presence of 5% CO_2_.

MG132 (Millipore Sigma, Burlington, MA, USA, Catalog # M8699-1MG) was reconstituted in DMSO to a stock concentration of 1 mM. Cells were treated with a final concentration of 10 μM added directly to the media where indicated.

HCMV strains PT36, a low-passage clinical isolate (kind gift of the late Dr. Nell Lurain, Rush University) and TB40/E-GFP [21] were used for these studies. HCMV infection was performed following overnight serum starvation at a multiplicity of infection (MOI) of 1.4 PFU/cell, a commonly used range for epithelial cell infection that enables synchronized infection while minimizing cytopathic effects that could nonspecifically impact host protein expression. Cells were harvested at 48 h post infection, a time point selected to evaluate early virus-induced changes in hormone receptor expression after establishment of immediate–early and early viral events but before the development of widespread cytopathic effects. Virus stocks were propagated in confluent ARPE-19 monolayers infected at a low MOI (0.001 PFU/cell) to permit multiple rounds of viral replication and generation of high-titer virus stocks. Cultures were monitored for 21 days or until the appearance of widespread cytopathic effects (CPE > 90%). Infected cells were then harvested by scraping with a cell scraper into the culture media, frozen at −80 °C, and thawed at room temperature. The suspension was then centrifuged at 1000 rpm for 10 min, supernatant was aliquoted, and the pellet was discarded. Virus stocks were stored at −80 °C. Virus titer was determined by plaque titer using NuFF cells as described [22]. For UV-inactivation, virus was treated with 120 mJ/cm^2^ of UV light (465 nm) using the UV Crosslinker FB-UVXL-1000 (Fisher Scientific, Waltham, MA, USA) and its Optimal Crosslink setting.

### 2.2. Immunoblotting

Cells were harvested using trypsin-EDTA (0.25%) with phenol red (Gibco, Billings, MT, USA, Catalog # 15400054) at 37 °C for 5 min, then the trypsin was neutralized by the addition of complete DMEM. The cells were then pelleted by centrifugation, washed with PBS, and the pellet was then suspended in lysis buffer (150 mM NaCl, 1%Triton x-100, 50 mM Tris-HCl pH 7.5, 0.1% sodium dodecyl sulfate (SDS)) supplemented with Protease Inhibitor Cocktail according to manufacturer specifications (Cell Signaling Technology, Danvers, MA, USA, Catalog # 5871).

Protein concentrations in each cell lysate were determined using the Bicinchoninic acid (BCA) protein assay (Thermofisher Scientific, Waltham, MA, USA, Catalog # 23225) and normalized to a concentration of 1.33 µg/µL. Following addition of 6X Reducing Laemmli Buffer (Thermofisher, Catalog # J61337-AC), the lysates were heated to 95 °C for 5 min. Lysate volumes of 20 µL were loaded onto 10% Mini-PROTEAN^®^ TGX™ Precast Protein Gels (Bio-Rad, Hercules, CA, USA, Catalog # 4561033) and electrophoresed in Tris-Glycine-SDS buffer at 70 V for two hours. Separated proteins were then transferred onto a 0.2 μm polyvinylidene difluoride (PVDF) membrane in transfer buffer using the Trans-Blot Turbo Blotting system (Bio-Rad, Catalog # 1704272). Membranes were then blocked for 1 h at room temperature using 5% non-fat milk in Tris-buffered saline with 0.01% Tween-20 (TBS-T). Membranes were incubated with primary antibodies (1:1000 dilution) in 0.5% milk in TBS-T overnight at 4 °C. The following primary antibodies were used for immunoblotting: rabbit anti-ER-α (Cell Signaling, Catalog # 13258S), rabbit anti-PR A/B (Cell Signaling, Catalog # 8757S), mouse anti-IE1/2 (Abcam, Cambridge, UK, Catalog # ab53495), mouse anti-β-Actin (Cell Signaling, # 3700S). After three washes for 10 min each in TBS-T, membranes were incubated with the horseradish peroxidase-conjugated anti-rabbit antibody (Cell Signaling, Catalog # 7074) or anti-mouse antibody (Cell Signaling, Catalog # 7076S) secondary antibody diluted 1:1000 in 0.5% nonfat milk in TBS-T for 1 h at room temperature. After washing in TBS-T, membranes were developed using Bio-Rad’s Clarity Western ECL Substrate (Bio-Rad, Catalog # 1705060) according to manufacturer instructions with the Bio-Rad ChemiDoc Touch imaging system. Representative images from three independent biological experiments are shown.

### 2.3. Immunofluorescence Microscopy

MCF-7 cells were seeded onto sterile, serum-conditioned glass coverslips placed in 6-well tissue culture plates to adhere for 12 h. Following serum starvation overnight, cells were mock infected or infected with HCMV strain TB40/E-GFP. At 48 h post-infection (hpi), cells were washed with phosphate-buffered saline (PBS) and fixed with 4% paraformaldehyde for 15 min at room temperature. Following fixation, cells were permeabilized with 0.1% Triton X-100 in PBS for 30 min then blocked in 5% bovine serum albumin (BSA) in PBS for 1 h at room temperature. Cells were incubated overnight at 4 °C with rabbit anti-ERα antibody (1:100 dilution), followed by washing with PBS and incubation for 1 h at room temperature with Alexa Fluor 647-conjugated goat anti-rabbit IgG secondary antibody (Cell Signaling, Catalog # 4414; 1:500 dilution). Coverslips were washed three times with PBS and mounted onto glass microscope slides using ProLong™ Gold Antifade Mounting reagent (Thermofisher, Catalog # P36941). Images were acquired using a Lionheart FX automated fluorescence microscope (BioTek Instruments, Winooski, VT, USA) controlled with Gen5 Image Prime software version 5.3.18 (BioTek Instruments). Images were collected using identical exposure times and acquisition settings for all experimental groups. ImageJ software version 1.54k (National Institutes of Health, Bethesda MD, USA) was used to quantify fluorescence intensity. Representative images from three independent biological experiments are shown.

### 2.4. RNA Extraction

Cells were harvested via trypsinization as described above. The cell pellet was then washed with phosphate-buffered saline (PBS), then centrifuged for 5 min at 1000 rpm. RNA was isolated using the Qiagen RNeasy Micro Kit (Qiagen, Hilden, Germany, Catalog # 74104) as specified by the manufacturer. The resulting RNA was then treated with DNAse I (Invitrogen, Waltham, MA, USA, Catalog # 18047019) and stored at −80 °C.

### 2.5. Quantitative Reverse Transcriptase Polymerase Chain Reaction (qPCR)

Reverse transcription (RT) was performed using Superscript III (Thermofisher, Catalog # 18080093) following instructions provided by the manufacturer and starting each reaction with 300 ng of RNA. The resulting cDNA was then treated with RNAse H (Invitrogen, Catalog # 18021014). Both standard PCR and qPCR were performed with this cDNA. Standard PCR was performed using 2X Taq polymerase Master Mix (New England Biolabs, Ipswich, MA, USA, Catalog # M0270L) and 1 μL of cDNA amplified with a PCR program consisting of 95 °C for 30 s (s), and 35 cycles of 95 °C for 30 s, 55 °C for 30 s, and 68 °C for 30 s, followed by a single 5 min elongation at 68 °C. PCR products were then analyzed via electrophoresis using a 2% agarose gel containing SYBR safe gel stain (Invitrogen, Catalog # S33102). qPCR was performed using PowerUp™ SYBR™ Green Master Mix (Applied Biosystems, Waltham, MA, USA, Catalog # A25743) with 1 μL of cDNA in each reaction with the following parameters: 95 °C for 10 min and 40 cycles of 95 °C for 15 s and 60 °C for 1 min. qPCR data were quantified from three independent biological experiments, each with three technical replicates, analyzed using the ΔΔCT method (where CT is cycle threshold) as described previously [23]. Fold change was calculated relative to mock infected control. The primers used in these experiments are as follows (5′ to 3′): *ESR1*(F)—AGCTTCGATGATGGGCTTAC, *ESR1*(R)—CACCATGCCCTCTACACATT, *PGR*(F)—ATTTCATCCACGTGCCTATCC, *PGR*(R)—CCTTCCTCCTCCTCCTTTATCT, GAPDH (F)—ACCACAGTCCATGCCATCAC, GAPDH(R)—TCCACCACCCTGTTGCTGTA. The PGR primer pair amplifies a coding region shared by both PR-A and PR-B transcripts and therefore quantifies total *PGR* expression rather than promoter-specific isoforms.

### 2.6. Statistical Analysis

Statistical analyses were performed using GraphPad Prism version 10 (GraphPad Software, Boston, MA, USA). Quantitative PCR experiments were performed using three independent biological replicates, each analyzed in triplicate technical replicates. Data are presented as the mean ± SEM from three independent biological experiments. Comparisons between mock- and HCMV-treated groups were performed using Welch’s unpaired *t*-test. Statistical significance was defined as *p* < 0.05. Exact sample sizes are provided in the corresponding figure legends.

## 3. Results

### 3.1. HR Levels Are Reduced During HCMV Infection

**Figure 1:**
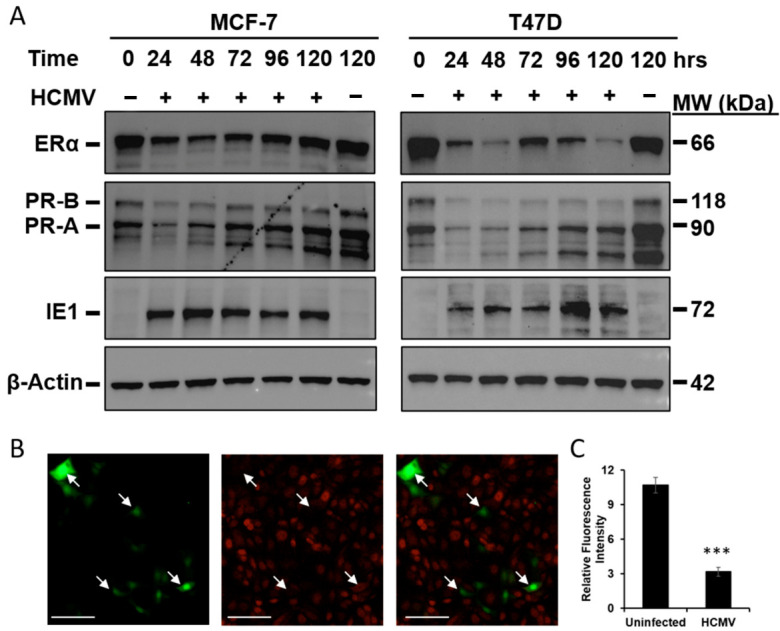
HCMV infection reduces hormone receptor levels in breast cancer cells. (**A**) Cells were harvested at the indicted times post infection and immunoblotted for human hormone receptors, HCMV IE1, and β-actin. These images are representative of three independent experiments. (**B**) MCF-7 cells were infected with HCMV TB40/E-GFP for 48 h and stained for ERα followed by Alexa 647 secondary antibody. Arrows highlight infected cells with low ERα levels. Scale bar = 100 µm. (**C**) ERα fluorescence intensity (red) quantified using ImageJ software for 10 cells/field infected with HCMV (green) and 10 uninfected cells/field (red only). Error bars, standard error; *** *p* = 0.0001, Welch’s *t* test.

HRs are critical diagnostic markers and therapeutic targets in breast cancer. To determine whether HR levels were impacted by HCMV infection, two different ER+/PR+ breast cancer cell lines, MCF-7 and T47D, were infected. Cells were harvested at 24 h intervals post-infection, and ERα and PR-A/B protein levels were determined by immunoblotting (Figure 1A). ERα levels in MCF-7 cells were markedly reduced at 24 hpi. ERα levels were further reduced at 48 hpi and then climbed steadily back up by 120 hpi. A similar pattern was observed in T47D cells, with maximum reduction in ERα around 48 hpi, and increased by 120 hpi. PR-A and PR-B exhibited a similar pattern of reduced expression at 24 and 48 hpi and then levels increased as the infection proceeded to 120 hpi. Notably, PR-A and PR-B did not return to starting levels by 120 hpi, but this was observed only in T47D cells. The IE1 protein is a marker for HCMV infection, and β-Actin served as a loading control. Overall, these results indicate that HCMV infection causes a transient decrease in HR levels in breast cancer cells.

To visualize hormone receptor expression at the single-cell level, MCF-7 cells were infected with the GFP-expressing HCMV strain TB40/E-GFP and immunostained for ERα. Consistent with the immunoblotting results, ERα immunofluorescence was markedly reduced in GFP-positive infected cells compared with mock-infected controls (Figure 1B,C).

**Figure 2:**
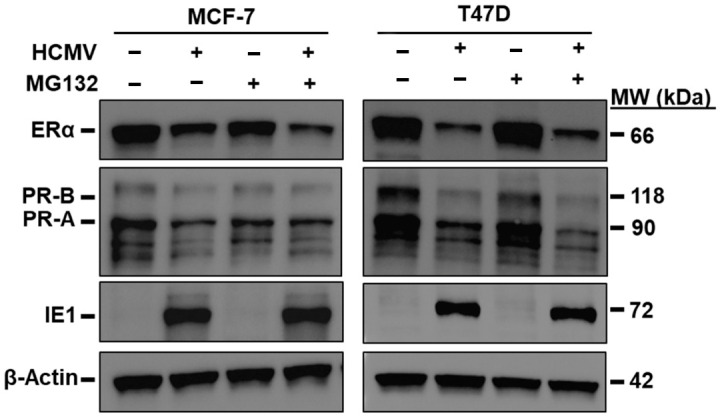
Proteasome inhibition does not restore hormone receptors in HCMV-infected cells. MCF-7 and T47D cells were mock- or HCMV-infected and harvested at 48 hpi. Cells were treated with MG132 (10 μM) for the final 6 h prior to harvest. Lysates were analyzed by immunoblotting for ERα and PR. Results shown are representative of three independent biological experiments.

We next explored potential mechanisms underlying HR downregulation induced by HCMV infection. We first considered the possibility that the HRs were being degraded by the ubiquitin-proteasome system. We predicted that blocking proteasomal activity would result in a recovery of HR levels in HCMV-infected cells. To explore whether ERα and PR-A/B were subject to proteasomal degradation during HCMV infection, cells were treated with the proteasomal inhibitor MG132. MCF-7 and T47D cells were infected for 48 h and 10 µM MG132 was added for the last six hours. Cell lysates were then immunoblotted (Figure 2). We observed that HR levels were still reduced during infection with no recovery of ERα and PR-A/B upon treatment with MG132, suggesting that proteasomal degradation was unlikely to be the primary mechanism for reduced HR levels observed in HCMV infected breast cancer cells.

### 3.2. HR Gene Expression Is Reduced During HCMV Infection

**Figure 3:**
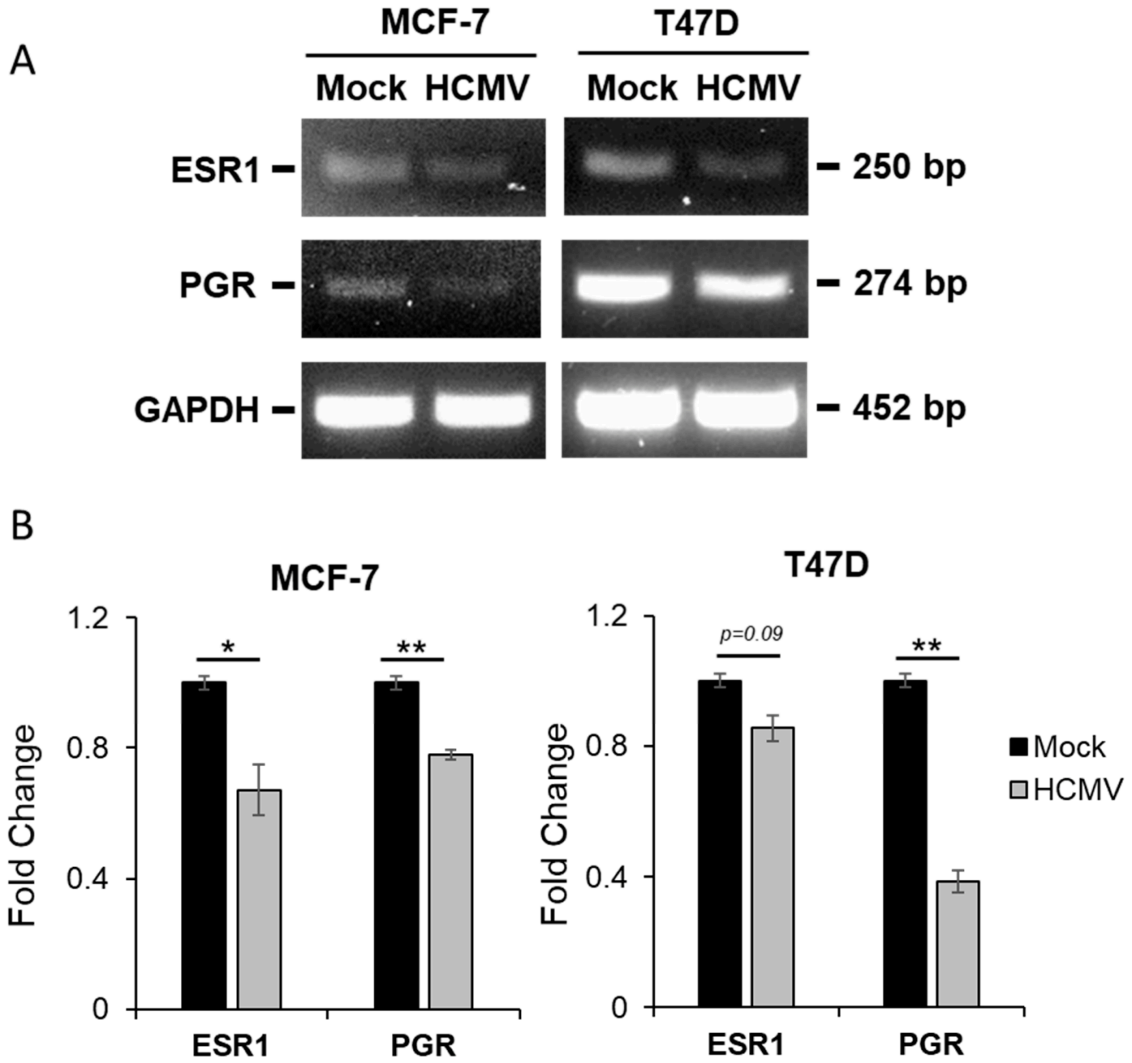
*ESR1* and *PGR* expression decreases following HCMV infection. (**A**) RT-PCR or (**B**) quantitative PCR (qPCR) was performed using RNA isolated from mock- and HCMV-infected cells harvested at 48 hpi. Gene expression levels of *ESR1* and *PGR* were normalized to GAPDH. Statistical analysis was performed using Welch’s *t*-test (n = 3 independent experiments). Error bars represent standard error of the mean (SEM). * *p* = 0.03; ** *p* = 0.009.

We investigated whether HR downregulation during HCMV infection occurred at the transcriptional level. To learn whether HCMV infection results in reduced expression of genes encoding HRs ERα (*ESR1*) and PR-A/B (*PGR*), we evaluated mRNA levels. We first performed standard RT-PCR (Figure 3A) using RNA extracted from MCF-7 and T47D cells at 48 hpi. Bands were visualized by agarose gel electrophoresis, and we observed decreased band intensity in HCMV-infected cells for both *ESR1* and *PGR*. qPCR was performed to measure this reduction in gene expression (Figure 3B). The results indicate that both *ESR1* and *PGR* expression were significantly decreased in HCMV-infected MCF-7 cells. For T47D cells, the decrease in *PGR* was significant, but the decrease in *ESR1* did not achieve statistical significance (*p* = 0.09), despite a qualitative reduction in band intensity observed by RT-PCR (Figure 3A).

### 3.3. Viral Gene Expression Is Not Required for HCMV-Induced Reduction in HR Levels

**Figure 4:**
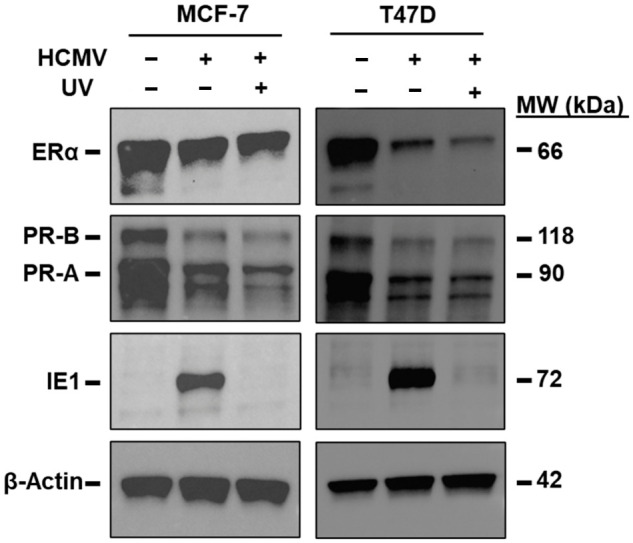
Virus replication is not required for hormone receptor reduction. MCF-7 and T47D cells were mock-infected, infected with HCMV, or treated with UV-inactivated HCMV and harvested at 48 hpi. Whole-cell lysates were analyzed by immunoblotting for ERα and PR. Data shown are representative of three independent biological experiments.

To determine whether viral gene expression is required to reduce HR levels, we treated cells with UV-inactivated virus. Exposure to UV light damages viral DNA, preventing viral gene expression and replication, yet previous studies have indicated that UV-inactivated virus particles may trigger intracellular immune responses, resulting in a cellular response to infection [24,25,26,27]. MCF-7 and T47D cells were infected with wild type or UV-inactivated HCMV for 48 h. Immunoblotting of lysates from these cells revealed a reduction in ERα and PR-A/B protein levels occurred whether cells were exposed to wild type, infectious HCMV or UV-inactivated virus, compared to levels in mock infected cells (Figure 4). The effectiveness of UV- inactivation was confirmed by the absence of bands corresponding to CMV IE1. These results suggest that viral gene expression is not required for the observed reduction in hormone receptor expression under these experimental conditions.

## 4. Discussion

HR -positive breast cancer accounts for the majority of newly diagnosed cases and is driven by estrogen and progesterone signaling pathways that are central to both tumor biology and therapeutic targeting [28]. In this study, we show that exposure to human cytomegalovirus (HCMV) is associated with a transient but marked reduction in ERα and PR expression in two independent breast cancer cell lines. This effect was observed at both the protein and transcript levels and occurred in the absence of productive viral replication, suggesting that early events associated with viral exposure are sufficient to alter HR regulation.

For patients with hormone receptor-positive breast cancer, endocrine therapies, including aromatase inhibitors and selective estrogen receptor degraders, are the standard of care. However, intrinsic and acquired endocrine resistance remain major clinical challenges [19,29,30,31,32,33]. Our findings demonstrate that HCMV exposure transiently reduces ERα and PR expression, raising the possibility that viral infection may influence endocrine therapy responsiveness. Functional studies directly evaluating treatment response will be required to determine whether HCMV-mediated hormone receptor suppression alters sensitivity to endocrine therapies.

Importantly, prior studies have demonstrated an inverse relationship between HCMV protein expression and hormone receptor levels in breast tumor tissues, supporting the potential in vivo relevance of our findings [18]. However, there is currently a lack of consensus as to whether evidence of HCMV infection is associated with ERα and PR levels in breast tumor biopsies [2,34]. This discrepancy could be explained by the heterogeneity of breast cancer cells, as the degree to which specific HRs are affected by HCMV in our study was dependent on cell type. As shown in Figure 1, ERα and PR are differentially affected in MCF-7 compared to T47D cells. Although both are mammary epithelial cells, they differ in their energetic properties and HR levels, and at least 164 proteins are differentially expressed between the two cell lines [35,36,37]. The differences observed between these well-characterized cell lines highlight the biological diversity of hormone receptor-positive breast cancers, yet HCMV consistently reduced hormone receptor expression in both models. Nevertheless, because MCF-7 and T47D cells are long-established breast cancer cell lines, they do not fully recapitulate the cellular heterogeneity and microenvironment of primary breast tumors. Validation of these findings in primary breast epithelial cells, patient-derived organoids, ex vivo breast tumor specimens, or in vivo models will therefore be important to determine the physiological and clinical relevance of HCMV-mediated hormone receptor suppression.

Transcriptomic evidence from human studies supports the concept that HCMV infection broadly alters estrogen-associated signaling pathways [38]. In a recent RNA-Seq analysis of peripheral blood mononuclear cells from renal transplant recipients with active HCMV viremia, estrogen signaling was identified as a significantly deregulated pathway, accompanied by downregulation of heparin-binding epidermal growth factor-like growth factor (HBEGF), a mitogen involved in estrogen and EGFR signaling crosstalk [38]. Although this study examined immune cells rather than breast epithelial cells, it provides independent in vivo evidence that HCMV can alter estrogen-associated signaling pathways. Together with our findings, these results support the concept that HCMV broadly modulates estrogen-responsive pathways across multiple cell types.

We found that treatment of MCF-7 and T47D cells with UV-inactivated virus still results in a robust decrease in hormone receptor levels. The observation that UV-inactivated HCMV produces a comparable reduction in ERα and PR suggests that viral gene expression and productive replication do not appear to be required for this effect. These findings suggest that early events associated with viral entry or virion-associated components are sufficient to alter hormone receptor expression. An important limitation of these experiments is that virus stocks were generated in ARPE-19 cells and therefore may contain host- or virus-derived soluble factors released during infection. Although our findings demonstrate that productive viral replication is not required for hormone receptor suppression, they do not formally distinguish effects mediated by virion-associated components from those of soluble factors present in the virus inoculum. Nevertheless, because hormone receptor downregulation was observed under conditions that did not produce extensive cytopathic effects, the response is unlikely to reflect a nonspecific consequence of high viral burden or widespread cellular damage. Experiments using purified virions and inoculum-matched controls will help distinguish virion-associated effects from soluble mediators and further define the signaling mechanisms responsible for hormone receptor suppression.

These findings are consistent with previous observations that actively replicating HCMV is not required is surprising, but not unfounded. Because UV-inactivation does not hinder viral entry or immune responses against viral particles, it is possible that viral proteins can still activate intracellular signaling pathways that ultimately result in HR reduction [27,39]. For example, the viral tegument protein pp71 can affect the cell cycle, and ERα expression fluctuates with phases of the cell cycle [40,41]. In fact, during cell cycle synchronization, ERα is most highly expressed during S phase [41]. It is also possible that downstream signaling from secreted factors such as Type I and Type II interferons from infected cells may be responsible for downregulating HRs [42,43].

Our findings indicate that HCMV-mediated hormone receptor downregulation occurs primarily through transcriptional suppression rather than enhanced proteasomal degradation. Quantitative PCR demonstrated significant suppression of *ESR1* transcripts in HCMV-infected MCF-7 cells and *PGR* transcripts in both MCF-7 and T47D cells (Figure 3), demonstrating that viral infection reduces HR expression at the transcriptional level. These findings are consistent with prior in situ hybridization studies showing reduced *ESR1* and *PGR* expression in regions of breast tumors with high viral protein abundance [18]. In parallel, pharmacologic inhibition of the proteasome using standard dosing of MG132 [44,45] failed to restore ERα or PR protein levels in HCMV-infected cells (Figure 2), further arguing against proteasome-dependent degradation as the primary mechanism of receptor loss. Although ERα and PR are known to undergo proteasomal turnover [46,47], proteasome inhibition can also induce compensatory autophagy, particularly in virally infected cells where increased metabolic and biosynthetic demands accompany viral replication [48]. Collectively, these findings support transcriptional suppression, rather than enhanced proteasomal degradation, as the primary mechanism of hormone receptor loss. While the present study establishes a replication-independent effect of HCMV exposure on hormone receptor expression, the upstream signaling pathways responsible for this transcriptional suppression remain to be defined. Candidate mechanisms include virion-associated proteins, early host pattern-recognition receptor signaling following viral attachment or entry, activation of inflammatory signaling pathways such as NF-κB, interferon- or MAPK/STAT-dependent signaling, epigenetic regulation of ESR1 and PGR transcription, and virus-induced alterations in transcription factor activity. In particular, SP1 and USF-1 are established regulators of the *ESR1* promoter [49], and HCMV has previously been shown to modulate SP1-dependent transcriptional pathways during infection [50,51], making these attractive candidates for future investigation. In addition, because the qPCR assay quantified total *PGR* transcripts, the present study could not determine whether HCMV differentially regulates the individual promoters responsible for PR-A and PR-B expression. Together, these findings provide a framework for defining the upstream signaling events and promoter-specific regulatory mechanisms by which HCMV suppresses hormone receptor expression in breast cancer cells.

Notably, while ER status is central to breast cancer classification and therapeutic decision-making, standard clinical assessment does not distinguish between ERα and ERβ, nor does it routinely evaluate the expression of the G protein-coupled estrogen receptor (GPER) [52]. This distinction may be particularly relevant in the context of viral modulation of HR expression. In this study, we demonstrate that HCMV exposure leads to a transient but significant downregulation of ERα and PR at the transcriptional level, independent of viral replication. Such selective suppression of ERα raises the possibility that estrogen signaling in infected cells may shift toward alternative receptor pathways, including ERβ or non-canonical signaling through GPER.

GPER mediates rapid, non-genomic estrogen signaling through G protein-dependent pathways and has been implicated in breast cancer progression, endocrine resistance, and estrogen responsiveness in ER-low or ER-negative tumors [52]. Importantly, several endocrine therapies developed to antagonize ERα have been shown to act as functional agonists of GPER [53], highlighting the complexity of estrogen signaling in tumors with reduced ERα expression. Although ERβ and GPER were not directly assessed in this study, our data demonstrate that HCMV infection selectively represses ERα expression, with downstream effects on progesterone receptor levels. Given that ERα directly regulates *PGR* transcription [54], this pattern suggests that viral modulation of ERα may be a primary event that reshapes hormone receptor signaling. Consistent with this model, our time-course analysis in MCF-7 cells shows early suppression of ERα in the absence of PR downregulation (Figure 1). Together, these findings identify HCMV-mediated suppression of hormone receptor expression as a previously unrecognized mechanism that may influence breast cancer biology and provide a foundation for future studies examining the signaling pathways responsible for this effect and its consequences for endocrine therapy responsiveness.

## 5. Conclusions

HCMV is a ubiquitous herpesvirus that has been detected in breast tumors, although its biological relevance remains incompletely understood. Here, we report that HCMV exposure is associated with suppression of hormone receptor expression in breast cancer cells independent of productive virus replication, suggesting that virus-associated signaling or host responses may contribute to altered hormone receptor regulation. Taken together, these findings identify HCMV as a previously unrecognized modulator of hormone receptor expression in breast cancer and support a model in which viral exposure suppresses ERα and PR through transcriptional mechanisms that do not require productive viral replication.

## Data Availability

The data presented in this study are available within the article. Additional data are available from the corresponding author upon reasonable request.

## Abbreviations

The following abbreviations are used in this manuscript:

ARPE-19	Adult Retinal Pigment Epithelial-19 cells
BCA	Bicinchoninic Acid
cDNA	Complementary Deoxyribonucleic Acid
CPE	Cytopathic Effects
DMEM	Dulbecco’s Modified Eagle Media
EDTA	Ethylenediaminetetraacetic Acid
EGFR	Epidermal Growth Factor Receptor
ERα	Estrogen Receptor-α
*ESR1*	Estrogen Receptor 1 (gene)
GAPDH	Glyceraldehyde 3-Phosphate Dehydrogenase
GPER	G Protein-Coupled Estrogen Receptor
HBEGF	Heparin-Binding Epidermal Growth Factor-Like Growth Factor
HCMV	Human Cytomegalovirus
HEPES	4-(2-Hydroxyethyl)-1-Piperazineethanesulfonic Acid
hpi	Hours Post-Infection
HR	Hormone Receptor
IE1	Immediate Early 1
MOI	Multiplicity of Infection
mRNA	Messenger Ribonucleic Acid
NuFF	Neonatal Foreskin Fibroblasts
PBS	Phosphate-Buffered Saline
PCR	Polymerase Chain Reaction
PFU	Plaque-Forming Units
*PGR*	Progesterone Receptor (gene)
PR	Progesterone Receptor
PVDF	Polyvinylidene Difluoride
qPCR	Quantitative Polymerase Chain Reaction
RNA	Ribonucleic Acid
RT	Reverse Transcription
SDS	Sodium Dodecyl Sulfate
SEM	Standard Error of the Mean
TBS-T	Tris-Buffered Saline with Tween-20
UV	Ultraviolet
